# NIR-Absorbing Mesoporous Silica-Coated Copper Sulphide Nanostructures for Light-to-Thermal Energy Conversion

**DOI:** 10.3390/nano12152545

**Published:** 2022-07-24

**Authors:** Elisabetta Fanizza, Rita Mastrogiacomo, Orietta Pugliese, Alexa Guglielmelli, Luciano De Sio, Rachele Castaldo, Maria Principia Scavo, Mariangela Giancaspro, Federica Rizzi, Gennaro Gentile, Fabio Vischio, Livianna Carrieri, Ilaria De Pasquale, Giacomo Mandriota, Francesca Petronella, Chiara Ingrosso, Marino Lavorgna, Roberto Comparelli, Marinella Striccoli, Maria Lucia Curri, Nicoletta Depalo

**Affiliations:** 1 Department of Chemistry, University of Bari, A. Moro, Via Orabona 4, 70126 Bari, Italy; r.mastrogiacomo@ba.ipcf.cnr.it (R.M.); oriettapugliese44@gmail.com (O.P.); mariangela.giancaspro@uniba.it (M.G.); federica.rizzi@uniba.it (F.R.); marialucia.curri@uniba.it (M.L.C.); 2 CNR-IPCF Bari Division, Via Orabona 4, 70125 Bari, Italy; f.vischio@ba.ipcf.cnr.it (F.V.); i.depasquale@ba.ipcf.cnr.it (I.D.P.); g.mandriota@ba.ipcf.cnr.it (G.M.); c.ingrosso@ba.ipcf.cnr.it (C.I.); r.comparelli@ba.ipcf.cnr.it (R.C.); m.striccoli@ba.ipcf.cnr.it (M.S.); 3 Department of Physics, NLHT-Lab, University of Calabria, Via Ponte P. Bucci, Cubo 33C, 87036 Rende, Italy; alexa.guglielmelli@unical.it; 4 CNR-NANOTEC-Institute of Nanotechnology, Via Ponte P. Bucci, Cubo 33C, 87036 Rende, Italy; luciano.desio@uniroma1.it; 5 Department of Medico-Surgical Sciences and Biotechnologies, Research Center for Biophotonics, Sapienza University of Rome, Corso della Repubblica 79, 04100 Latina, Italy; 6 CNR-IPCB, Via Campi Flegrei 34, 80078 Pozzuoli, Italy; rachele.castaldo@ipcb.cnr.it (R.C.); gennaro.gentile@ipcb.cnr.it (G.G.); 7 National Institute of Gastroenterology Saverio de Bellis, IRCCS Research Hospital, Via Turi 27, 70013 Castellana Grotte, Italy; maria.scavo@irccsdebellis.it (M.P.S.); livianna.carrieri@irccsdebellis.it (L.C.); 8 CNR-IC, Via Salaria Km 29.300, 00015 Monterotondo, Italy; francesca.petronella@ic.cnr.it; 9 CNR-IPCB, Piazzale E. Fermi 1, 80055 Portici, Italy; marino.lavorgna@cnr.it

**Keywords:** plasmonic nanostructures, Cu_2−x_S nanocrystals, mesoporous silica, photothermal properties

## Abstract

Plasmonic nanostructures, featuring near infrared (NIR)-absorption, are rising as efficient nanosystems for in vitro photothermal (PT) studies and in vivo PT treatment of cancer diseases. Among the different materials, new plasmonic nanostructures based on Cu_2−x_S nanocrystals (NCs) are emerging as valuable alternatives to Au nanorods, nanostars and nanoshells, largely exploited as NIR absorbing nanoheaters. Even though Cu_2−x_S plasmonic properties are not linked to geometry, the role played by their size, shape and surface chemistry is expected to be fundamental for an efficient PT process. Here, Cu_2−x_S NCs coated with a hydrophilic mesoporous silica shell (MSS) are synthesized by solution-phase strategies, tuning the core geometry, MSS thickness and texture. Besides their loading capability, the silica shell has been widely reported to provide a more robust plasmonic core protection than organic molecular/polymeric coatings, and improved heat flow from the NC to the environment due to a reduced interfacial thermal resistance and direct electron–phonon coupling through the interface. Systematic structural and morphological analysis of the core-shell nanoparticles and an in-depth thermoplasmonic characterization by using a pump beam 808 nm laser, are carried out. The results suggest that large triangular nanoplates (NPLs) coated by a few tens of nanometers thick MSS, show good photostability under laser light irradiation and provide a temperature increase above 38 °C and a 20% PT efficiency upon short irradiation time (60 s) at 6 W/cm^2^ power density.

## 1. Introduction

In the past decades theoretical studies [1,2,3], as well as in vitro and in vivo investigation [4,5,6,7,8], have demonstrated the great potential of colloidal plasmonic nanoparticles (NPs) as nanoheaters in targeted photothermal (PT) therapy, and are suggested as an alternative approach to chemotherapy for cancer treatment [9,10,11]. Photoexcitation of the NP surface plasmon oscillations by a resonant laser light results in NP light absorption and, due to subsequent nonradiative relaxation, thermal energy is released through the NP interface into the environment [1,2]. Upon cellular uptake of the tumor targeted plasmonic NPs, they can promote a hyperthermia process thanks to a laser-induced temperature increase, that ultimately can damage and kill cancer cells with little or no harm to healthy tissues [12]. A similar laser light-activated process can be also investigated on plasmonic NPs that behave as exogenous contrast agents for non-invasive photoacoustic imaging (PAI). Upon excitation with appropriate short-pulsed light, NPs convert the absorbed incident photons into heat, generating a thermoacoustic wave, which can be detected by an ultrasound transducer for image reconstruction [3]. 

Considering the PT properties of the plasmonic nanostructures, an enhancement of light-to-thermal energy conversion efficiency requires their careful design in terms of morphology, composition and surface chemistry. These features may all affect the localized surface plasmon resonance (LSPR) band and the extinction cross-section, σ_extinction_, comprising the absorption, (σ_absorption_) and scattering (σ_scattering_) contributions. Higher PT efficiency is expected for NPs characterized by an extinction cross-section with a relevant absorption contribution and negligible scattering since the absorption cross-section governs the thermal transduction per particle. For Au NPs, which are the foremost plasmonic materials investigated in biomedical applications, the absorption efficiency and the σ_absorption_/σ_scattering_ ratio, in the entire visible spectrum (400–800 nm), evaluated by using the Mie-Gans theory, increase as the NPs size decreases. This suggests that the use of small plasmonic NPs can be advantageous for an effective light-to-heat conversion [11]. 

NP shape has been also proven to improve PT conversion [13,14]. In the case of Au NPs, the shape dependent LSPR results in an LSPR band located in the visible region for the nanosphere, that red-shifts to the first (I)- near-infrared (NIR) window (700–980 nm) for anisotropic shapes, nanoshells [4] or nanocages. The availability of NIR plasmonic nanostructures is promising for PT therapy, as it enables the use of NIR laser light to excite NP surface plasmons and promote absorption. Compared to visible light, the NIR radiation can penetrate deeper into biological tissues, with minimal attenuation by water and hemoglobin and other biological components, and thus, reduced interference due to cell constituents. Such features, in turn, enhance NP absorption, improve PT efficiency, and increase therapeutic efficacy for subcutaneous tumor treatments [1,3,15]. Moreover, the anisotropic plasmonic NPs are valuable in PT therapy not only for their NIR absorbing properties. Theoretical simulations [1,3,16] and experimental studies demonstrate an electrical field enhancement, occurring at the sharp edges or vertices in anisotropic NPs, due to hot spot generation, which is reflected by a strong increase in temperature [17]. Localization of the electric field intensity on the tips leads to a strong dephasing of coherently oscillated surface electrons, whose energy is transferred to the atomic lattice effusing a strong flux of heat at the particle−dielectric interface [18]. A large body of literature deals with the fabrication of anisotropic Au NPs, such as nanorods [1,12], nanostars [17], or spiky nanostructures [19] for in vitro and in vivo investigation of their PT properties, using minimally invasive NIR radiation as excitation. However, nanostructures, featuring high curvature (i.e., nanorods, nanostars, spiky NPs), present limited stability under prolonged light exposure, as they may undergo deformation and reshaping phenomena [19,20,21,22,23,24].

Another plasmonic NPs feature, that contributes to increase the efficiency of the PT process is their surface chemistry, since it affects the heat transfer process from the NP to the dispersant medium [2,13]. Colloidal plasmonic NPs are generally coated by either an organic (molecular or polymeric) or inorganic shell that confers colloidal stability. Such a coating acts as an intermediate layer between the plasmonic NPs and the environment and affects the heat diffusion into the dispersant medium [12]. The thermal conductivity of the NPs and their coating and their interfacial thermal resistance with respect to the surrounding solvent, control the rate of heat release. Several studies have been performed to evaluate heat diffusion from excited Au nanospheres [25] and nanorods [12,26], coated with molecular (i.r. cetyl trimethyl ammonium bromide), polymeric (i.e., polyethylene glycol or polyelectrolytes) or inorganic nonporous and mesoporous silica shell (MSS) [17,24]. In particular, systematic studies carried out on silica coated Au-nanostructures, have demonstrated the role of the plasmonic core size and shape, the silica shell thickness and texture [24] in tuning the PT properties of the systems. Even though the mechanism has not been clearly understood [24,26], the silica coating has been demonstrated to enhance the PT properties. Such an effect has been explained by taking into account the low interfacial thermal resistance between the Au core and the surrounding solvent in the presence of the silica shell, which allows fast heat transfer in the environment from the plasmonic NPs through the interface [24,26]. More recently, a theoretical investigation on Au–silica core-shell nanostructures, carried out to understand the impact of their structures in the enhanced response expected in PAI application, has concluded that direct electron–phonon coupling through the interface may provide another channel for heat to flow across the metal-dielectric interface, resulting in enhanced thermal transport at metal–nonmetal interfaces when such electron–phonon conductance is present [27,28]. Indeed, a deep understanding of the types of phenomena involved in the light-to-thermal energy conversion and heat transfer enhancement is strongly related to the nanostructure’s properties, including the core and shell geometry, and laser pulse duration. 

Furthermore, it has been found [24] that a thick shell preserves the PT material properties, providing improved protection of the Au core from photodegradation. However, the advantageous role of the silica shell arises only below a certain shell thickness threshold, of nearly 20 nm. In fact, above 20 nm scattering phenomena, which reduce absorption efficiency, have been found to markedly decrease the light-to-thermal energy conversion. 

To overcome the limitations of Au-based nanostructures as nanoheaters, such as poor photostability and shape-dependent NIR absorption, new plasmonic materials with large NIR absorption have been recently proposed as innovative alternatives to Au-NPs. In this regard, copper sulphide NPs, with the general formula, Cu_2−x_S, have emerged as viable NIR-absorbing plasmonic materials and recent studies have fostered their possible use in biomedical applications [29,30,31]. Less expensive than noble metals and more stable upon prolonged optical excitation, Cu_2−x_S is a p-type semiconductor with free hole carriers, whose density sustains an LSPR band covering the second(II)-NIR window (1000–1700), which is not bound to specific shapes but dependent on phase and stoichiometry [30,32,33,34,35,36,37,38,39].

In vitro and in vivo studies of the NIR-light induced PT activities of Cu_2−x_S nanocrystals (NCs) coated by hydrophilic molecular ligands [38,39,40] or a MSS [35,41] have been reported in the literature; however, to the best of our knowledge, the systematic investigation of the role of Cu_2−x_S NCs size and shape and of the impact of the surface chemistry/interface on the PT efficiency has been still not fully addressed. 

Here, core-shell silica coated Cu_2−x_S NCs, featuring different sized and shaped cores and a MSS, have been synthesized via solution-phase approaches as potential PT nanoagents. The Cu_2−x_S NCs core features the plasmonic properties, while the MSS may confer the core protection from the external environment and additional drug loading capability. Particles with such an architecture can simultaneously deliver the plasmonic NPs as well as additional active molecules, including contrast agents [42], for imaging-guided PT therapy, or chemotherapeutic agents, for achieving a synergistic treatment based on PT therapy and chemotherapy, as it has been demonstrated that their combination is able to enhance therapeutic efficacies and reduce unwanted side effects [43]. 

This study aims at optimizing the Cu_2−x_S NCs-based nanostructures characteristics, such as core morphology, shell thickness, and textural properties to enhance the light-to-heat conversion efficiency and heat transfer properties of the systems. Indeed, these characteristics have been already demonstrated for the Au-based plasmonic nanostructures and are essential for achieving photostable and photothermally efficient systems [44,45].

Here, organic-capped spherical Cu_2−x_S nanoparticles (NSs) and facet triangular nanoplates (NPLs), with an average diameter of 7 nm and edge length of 14 nm, respectively, have been prepared by the hot-injection strategy and coated with a hydrophilic MSS, purposely tuning its thickness and texture. A comprehensive spectroscopic, morphological, and structural characterization of each type of nanostructure has been completed with a systematic and fundamental analysis of the PT process. The experiment, performed by irradiating the aqueous dispersion of the core-shell NPs by a continuous wave (CW) pump beam (λ = 808 nm), for a short time (60 s), by properly tuning the laser beam density, resulted in temperature changes that have been monitored by means of a thermal camera. The study has led to the elucidation of the influence of Cu_2−x_S morphology and MSS characteristics, thickness and texture on the efficiency of the PT process. 

## 2. Materials and Methods

### 2.1. Materials 

For the synthesis of Cu_2−x_S NCs: Copper(I) chloride (CuCl, 99.99%), copper(II) chloride (CuCl_2_, 90%), sulphur powder (S8, 99.99%), 1-octadecene (ODE, technical grade 90%), oleic acid (OA, technical grade 90%), oleylamine (Olam, 70%), chloroform (CHCl_3_), ethanol (EtOH), tetrachloroethylene (TCE), acetone, methanol (MeOH) and isopropopanol (iPOH) were purchased from Sigma-Aldrich, at maximum purity available.

For the MSN and MSS: Cetyl trimethylammonium bromide (CTAB > 96%), NaOH, tetraethyl orthosilicate (TEOS 98%) absolute ethanol (EtOH, 98%), ethyl acetate, HCl (32% aqueous solution) were purchased from Sigma-Aldrich (Milan, Italy). All aqueous solutions were prepared by using water obtained from a Milli-Q gradient A-10 system (Millipore, Bedford, MA, USA) 18.2 MΩcm, organic carbon content ≥ 4 µg/L). For cell viability were used RBE and EGI cell lines purchased from ATCC (Manassas, VA, USA) and CellTiter 96 AQueous One Solution Cell Proliferation Assay (MTS) was purchased from Promega (Madison, WI, USA). 

### 2.2. Synthesis of Colloidal Cu_2−x_S Nanocrystals

Cu_2−x_S NCs with tailored morphologies were synthesized according to the procedures reported in Giancaspro et al. [46], based on the hot-injection method under air-free conditions using a standard Schlenk line setup. In a three-necked flask, 32 mg (1 mmol) of elemental sulphur and 2.5 mL of OA (4 mmol) were dispersed in 2.5 mL of ODE. In a second three-necked flask, the copper precursor was prepared by dispersing 0.5 mmol of CuCl (49.5 mg) or CuCl_2_ (67.2 mg), used for the synthesis of nanospheres (NSs) and triangular nanoplates (NPLs), respectively, and 1.5 mL (2.5 mmol) of OA and 3.5 mL (5 mmol) of Olam into 7.5 mL (10 mmol) of ODE. The two reaction flasks were first heated at 100 °C for 1 h under vacuum and vigorous magnetic stirring to remove air and moisture. The sulphur precursor reaction mixture was then heated up to 150 °C, under nitrogen for 1 h and then cooled down at 60 °C. The copper precursor flask was heated up to 180 °C under nitrogen and, after 15 min, 2.5 mL of the sulphur precursor stock solution (Cu:S precursor molar ratio 1:1) was swiftly injected and kept at 180 °C for 10 min. The reaction mixture was finally cooled down to room temperature, and the NCs were collected by addition of EtOH as non-solvent (reaction mixture: EtOH volume 1:3) and centrifugation at 950× *g* for 10 min (Centric 322 A, Tehtnica). A few drops of CHCl_3_ were added to the pellet followed by the addition of 5 mL of EtOH. The sample was then centrifuged again, and the final collected precipitate was redispersed in 2.5 mL of TCE for further characterization. NS and NPL concentration were 22 μM (18 mg/mL) and 25 μM (20 mg/mL), respectively. Molar concentration was determined from the absorbance spectrum at a known dilution, the concentration expressed in mg/mL was measured by weighting an aliquot of the solution after solvent removal under nitrogen flux. 

### 2.3. Synthesis of Mesoporous Silica Nanoparticles (MSN)

The mesoporous silica NPs (MSNs) were synthesized according to the microemulsion approach reported by Rizzi et al. [47] with minor modifications. Namely, CTAB (95 mg, 0.26 mmol) was dispersed in 50 mL of an alkaline aqueous solution by NaOH 5 mM, and 3 mL of ethyl acetate and 0.5 mL (2.2 mmol) of TEOS were added. The solution was kept under stirring at 40 °C for 2 h and then the temperature was increased up to 60 °C, for an overall time of 1 h, including the temperature ramp. Finally, the microemulsion was left for 24 h at room temperature (25 °C). The obtained suspension was centrifuged (13,000× *g* at 18 °C for 1 h), to recover the MSNs as a pellet, while unreacted precursors and surfactant in the supernatant were discarded. The pellet was redispersed in a solution of HCl at 0.16 M in ethanol (V = 20 mL) and sonicated for 3 h to remove the CTAB molecules still entrapped in the mesopores. Subsequently, the suspension was subjected to repeated cycles of centrifugation and redispersion in water and the final pellet was redispersed in 2 mL of Millipore H_2_O. The MSNs concentration, measured by freeze-drying an aliquot of the sample, was nearly 6 mg/mL.

### 2.4. Synthesis of Cu_2−x_S NCs Coated by a Mesoporous Silica Shell

For the MSS growth, the Cu_2−x_S NCs were preliminary surface treated by the fresh addition of the native OA and Olam ligands. In detail, 0.5 mL colloidal solution in TCE of the as-prepared Cu_2−x_S sample, either NS or NPL samples, OA 0.13 M and Olam 0.12 M were stirred for 1 h at room temperature. Then, TCE was partially removed under nitrogen flux and ethanol was added as a non-solvent to recover the NCs by centrifugation (13,000× *g* at room temperature for 10 min), and finally redispersed in 0.5 mL of CHCl_3_; 0.3 mL of this colloidal solution were diluted with CHCl_3_ up to 0.5 mL (13 μM and 15 μM for NS and NPL samples, respectively), and subsequently 5 mL of an aqueous solution of CTAB 55 mM were added. The obtained microemulsion was vigorously stirred for 1 h, prior to removing CHCl_3_ under vacuum, resulting in a clear pale-brown colloidal solution. It was poured into a 70 mL bottle and finally diluted with 45 mL of MilliQ water. To the aqueous colloidal solution, 3 mL of ethyl acetate and variable volume of TEOS, namely 0.3 mL of TEOS (1.3 mmol) or 0.5 mL (2.2 mmol), were added for the preparation of thinner (NPL@MSS_03 or NS@MSS_03) or thicker (NPL@MSS_05 or NS@MSS_05) MS, respectively. The bottle was sealed with a rubber septum, put under a nitrogen atmosphere by bubbling nitrogen via a needle, while continuously stirring and then 0.1 mL of NaOH solution (0.1 g/mL, 2.5 M) was injected to reach a NaOH concentration of 5 mM in the reaction mixture. Then, the bottle was put in a heating bath set at 40 °C and heated for 2 h, followed by 1 h at 60 °C, as previously described for the MSN, under continuous stirring. Then the reaction mixtures were cooled down to room temperature and left under stirring overnight. 

The samples were purified by repeated cycles of centrifugation/redispersion in ethanol (13,000× *g* at 18 °C for 1 h) to remove unreacted precursors and surfactant and finally treated with an alcoholic acidic solution of HCl in EtOH at 0.16 M (V = 20 mL), sonicated for 3 h to remove the CTAB micelles entrapped in the mesopores and finally centrifuged to recover the NPs as a pellet that was redispersed in 2 mL of Millipore H_2_O. The concentration of each core-shell sample was measured by freeze-drying an aliquot of the sample, resulting in nearly 15 mg/mL for those samples prepared by using 0.5 mL of TEOS, either NPL@MSS_05 or NS@MSS_05, and 6 mg/mL for those obtained by using 0.3 mL of TEOS, either NPL@MSS_03 or NS@MSS_03.

### 2.5. Characterization Techniques

UV-Vis-NIR absorption spectra of Cu_2−x_S NCs, MSN and core-shell samples were measured in 1 cm path length matched paired quartz cuvettes using a Cary Varian 5000 spectrophotometer supplied with a double detector. Cu_2−x_S NCs were diluted by 1:250 for the UV-Vis-NIR spectroscopic characterization using TCE as the dispersant solvent, which is transparent in the NIR spectral range. Spectra of the MSN and core-shell nanostructures were recorded in ethanol by diluting the samples 1:2. 

TEM measurements were performed by a JEOL JEM1011 (JEOL Akishima, Tokyo, Japan) electronic microscope operating at 100 kV, equipped with a high-resolution CCD camera, using carbon-coated copper grids. The TEM grids were dipped in the Cu_2−x_S NCs colloidal solution diluted by 1:250 with TCE letting the solvent evaporate. The MSN or Cu_2−x_S@MSS samples were prepared for TEM imaging by drop-casting 3 μL of each suspension on a TEM grid followed by air-drying. Statistical analysis of the TEM images was performed using the ImageJ analysis freeware, and the average diameter of the Cu_2−x_S NSs, MSNs and core-shell nanostructures and edge-length of Cu_2−x_S NPLs were determined by measuring nearly 150 particles and their size distribution calculated as percentage relative standard deviation (σ%). 

Energy Dispersive X-ray (EDX) spectroscopy was carried out using a Field Emission Sigma Zeiss SEM microscope equipped with a LaB_6_ source thermal field emitter, working at 20 keV as acceleration voltage and 7 mm as working distance. The Cu_2−x_S NCs colloidal solutions were cast on a freshly cleaned polished silicon substrate. Silicon substrates with a dimension of 15 × 15 mm^2^ were cleaned following three successive washing cycles by sonication in MeOH and acetone for 5 min, alternately, interspersed with immersion in iPOH. Then 50 μL of dispersion of the as-synthesized Cu_2−x_S NPL and NS samples were drop cast on the silica substrate. The samples were mounted on a metal stub with conductive adhesive and EDX spectra and elemental analysis were acquired on three different area of the substrate.

For dynamic light scattering (DLS) and ζ- potential measurement, a Zetasizer nano ZSP (Malvern, Worcestershire, United Kingdom) equipped with a 50 mW laser diode emitting at 532 nm, was used. For the analysis, a solution of 7 μg/mL in filtered ultrapure water was used. 

Nitrogen adsorption analysis was performed to evaluate the textural properties of the Cu_2−x_S@MSS. Brunauer–Emmett–Teller (BET) specific surface area was evaluated by N_2_ adsorption at 77 K through an ASAP 2020 analyzer (Micromeritics, Norcross, GA, USA). Nitrogen adsorption analysis was performed up to 1 bar, and results were plotted to show the volume of nitrogen adsorbed per gram of material versus the relative pressure p/p^0^, where p represents the N_2_ absolute pressure and p^0^ represents the saturation pressure of N_2_ at the temperature of analysis (760 mmHg at 77 K). Pore size distribution was evaluated using the Non-local Density Functional Theory (NLDFT) analysis. The adsorption measurements were performed using high purity gases (>99.99%). The samples were degassed at 100 °C under vacuum before analysis (*p* < 10^−5^ mbar). 

### 2.6. Evaluation of Photothermal Properties

To investigate the PT properties of MSN and Cu_2−x_S NCs coated by MSS, a thermo-optical setup (Figure S1 Supplementary Materials) was used, based on a CW diode laser (Coherent Powerline) operating at 808 nm. Laser beam density in the range of 6–38 W/cm^2^ was tested. The PT measurements were performed by using a quartz cuvette that was filled with 1 mL of the investigated colloidal dispersions. A high-resolution thermal camera (FLIR, A655sc) was used for mapping and identifying both the spatial heating distribution and the temperature profile under lateral pumping laser irradiation. The camera produced thermal images of 640 × 480 pixels with an accuracy of ±0.2 °C. It worked seamlessly with proprietary software (FLIR ResearchIR Max) to record and process the acquired thermal data.

The PT conversion efficiency (*η*) was calculated using the Roper model [24,48]

$$\eta \;=\;\frac{\mathrm{hS}(T_{\max}\;-\;T_{\mathrm{sur}})\;-\;Q_{0}}{I(1\;-\;10^{-A_{808}})}$$

where *h* and *S* are the heat transfer coefficient and surface area of the quartz cuvette covered by the sample, respectively; *T_max_* and *T_sur_* are the maximum temperature under laser irradiation and the ambient temperature, respectively; *Q*_0_ represents the heat dissipated due to solvent absorption under the 808 nm laser irradiation and I and *A*_808_ are the incident laser power and absorbance of the solutions at 808 nm, respectively. To calculate the product *h S*, a dimensionless driving force temperature (*θ*) is introduced.

$$\theta \;=\;\frac{T(t)\;-\;T_{\mathrm{sur}}}{T_{\max}\;-\;T_{\mathrm{sur}}}$$

The sample system time constant, *τ*, can be calculated as follows with

$$-ln\theta \;=\;\frac{t}{\tau }$$

as the negative reciprocal slope of *ln*(*θ*) versus *t* using temperature versus time data recorded during cooling after the laser was switched off and

$$\mathrm{hS}\;=\;\frac{m_{s}\;-\;c_{s}}{\tau }$$

where *m_s_* and *c_s_* are the mass and heat capacity of the solution.

### 2.7. Cell Culture and Cell Proliferation Assay

Human cholangiocarcinoma cells namely RBE and EGI were purchased from ATCC. Both cell lines were cultivated according to retailer protocols. Briefly, for both cell lines, RPMI (GIBCO) supplemented with 10% of Inactivated Fetal Bovine Serum (FBS), sodium pyruvate, 4.5 g/L glucose, 4 mM L-Glutamine and 1% of Pen-Strep (penicillin 10,000 μ/mL, streptomycin 10,000 μ/mL, Lonza Biowhittaker) was used. Cells were grown until reaching semi-confluence, in a humidified incubator at 37 °C with an atmosphere containing 5% of CO_2_ and then used for the cell proliferation assay.

Cholangiocarcinoma cell lines, namely RBE and EGI, were seeded into 96-well plates at a density of 2 × 10^3^ cells per well and after 24 h were exposed to MSS and NPL@MSS_05, dispersed in water at concentrations ranging from 100 to 500 µg/mL, at 37 °C for, 24, 48 and 72 h. Controls were represented by untreated cells. After treatment, an MTS tetrazolium compound reagent (20 μL) for a total volume of 120 μL was added to the cells for 3 h at 37 °C and the absorbance at 490 nm was measured (PerkinElmer Victor Plate Reader, Belgium). Statistical analysis was performed by using a one-way analysis of variance (ANOVA) by SIGMA-STAT 3.5. In particular, when this analysis rejected the hypothesis of the mean equality among the groups, the Bonferroni methods were applied [49]. 

## 3. Results

### 3.1. Design of the Cu_2−x_S NCs and Mesoporous Silica Shell

**Figure 1:**
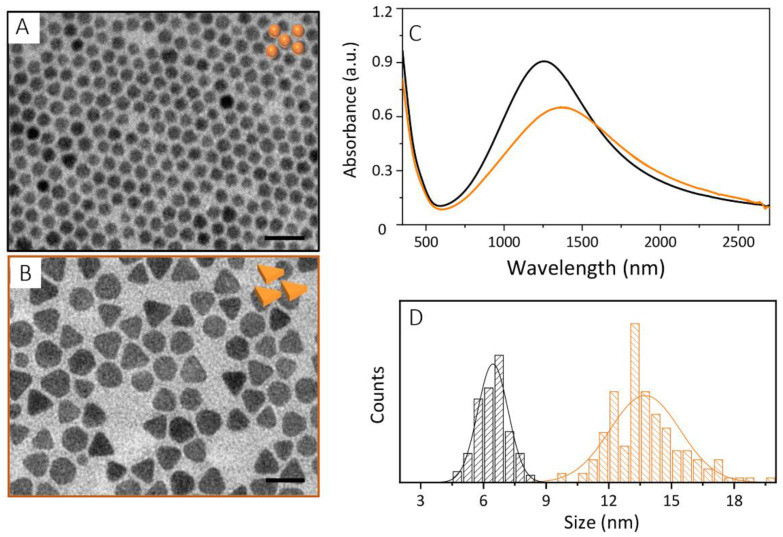
TEM micrographs ((**A**, **B**), scale bar 20 nm) and UV-Vis-NIR absorption spectra (**C**) of Cu_2−x_S nanocrystals synthesized by the hot-injection approach using CuCl (A, black line C) and CuCl_2_ ((**B**), orange line (**C**)) precursors. Panel (**D**), diameter (black bar) and edge-length (orange bar) distribution of Cu_2−x_S nanocrystals as measured by statistical analysis of TEM micrographs reported in panel A and in B, respectively.

The versatile solution-phase approaches in the synthesis of colloidal nanostructures have here provided viable tools and straightforward procedures for the fabrication of core-shell NPs based on Cu_2−x_S NCs, with tailored geometry, and MSS, with controlled thickness at the nanometer scale. Cu_2−x_S NCs featuring spherical and facet shapes have been synthesized by the hot-injection approach. As accounted by TEM characterization (Figure 1A,B), NSs (Figure 1A) with an average diameter of nearly 7 nm (σ% = 11, Figure 1D black histogram) and slightly larger triangular nanostructures (Figure 1B), ascribed to nanoplates (NPLs), with an average 14 nm edge-length (σ% = 12, Figure 1D, orange histogram) have been obtained, by simply tuning the reaction mixture composition. As described by Giancaspro et al. [46] starting from CuCl in an ODE/OA/Olam reaction mixture, small NSs, having an average size that decreases with a decrease in the reaction temperature (data not reported), are attained upon the injection of a sulphur precursor, obtained by the thermal decomposition of S_8_ in ODE/OA. When CuCl_2_ is exploited as a precursor, under the same reaction conditions and reaction mixture composition, facet NCs with a triangular section, and almost uniform in size and shape and ascribed to NPLs are achieved. Shape regulation has been attributed to the kinetics of monomer release and nucleation, due to the salt precursors’ reactivity, and to ion- and ligand-directed growth. Fast cuprous precursor decomposition and monomer formation rapidly burst nucleation that, according to LaMer theory, lead to smaller spherical NCs. 

Conversely, the monomer formation has slow kinetics, stalled by the cupric reduction to cuprous to form [CuS] monomer, using CuCl_2_ precursors, and results in NCs having a slightly larger size, and a final faceted morphology, directed by the selective adsorption of ligands and counter ions (chloride) on specific NC faces. UV-Vis-NIR absorption spectra (Figure 1C) recorded on both samples confirm the characteristic line profile of Cu_2−x_S NCs, showing a high absorption in the UV and blue-green region of the electromagnetic spectrum, ascribed to semiconductor behavior, and an intense band covering the I- and II-NIR window, attributed to the Cu_2−x_S LSPR band. A much broader and red-shifted LSPR band, centered at 1368 nm, (Figure 1C, orange trace) has been recorded for the NPLs, with respect to the NSs, that, instead, presents a more intense LSPR band located at 1263 nm (Figure 1C, black trace). Besides such differences in the LSPR band profile and position, both the NCs are digenite in phase, as demonstrated by X-ray characterization [46] performed on the samples and as confirmed by the NC stoichiometry estimation from the spectroscopic and morphologic investigation (see table in Figure S2A, in the Supplementary Materials). EDX analysis (Figure S2C,D in Supplementary Materials) on drop-cast samples confirms the presence of copper and sulphur elements. The elemental analysis returns a stoichiometry of Cu_1.84_S and Cu_1.78_S for Cu_2−x_S NPL and NS samples, respectively, which are close to what is expected from the digenite phase and predicted by UV-Vis-NIR absorbance spectra.

The organic-capped Cu_2−x_S NCs have been phase transferred in aqueous media by growing on an MSS, which confers hydrophilicity, protection of the core from the environment, and loading capability. Meanwhile, as demonstrated for similar nanostructures based on Au NPs coated with a silica-based shell, the silica network is expected to provide enhancement in light-to-thermal energy conversion and more effective heat dissipation in the suspended media rather than Cu_2−x_S NCs functionalized with hydrophilic polymers or molecular ligands [24]. 

**Figure 2:**
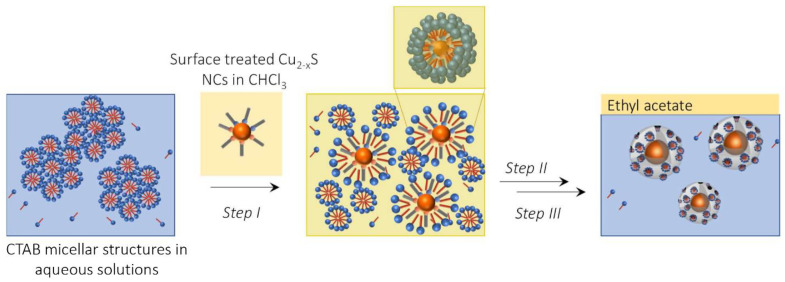
Scheme of the synthetic steps performed to grow the mesoporous silica shell coating the Cu_2−x_S nanocrystals.

Inspired by procedures reported in the literature [50,51,52,53], describing encapsulation in the MSS of organic-stabilized NCs as organic-capped Fe_2_O_3_ and CdSe quantum dots; an analogous strategy has been here developed to coat Cu_2−x_S NPLs and NSs with an MSS, using a variable volume of TEOS. A representative sketch of the synthetic procedure is reported in Figure 2. 

After a preliminary NC surface treatment with fresh Olam and OA ligands, aiming at restoring the ligands shell protecting the native NCs, without altering their plasmonic properties (See Figure S2B in the Supplementary Materials), the hydrophobic Cu_2−x_S NCs have been dispersed in a water solution of CTAB forming an oil-in-water microemulsion (Step I, Figure 2). The cationic CTAB plays a two-fold role, as it disperses in the aqueous environment the hydrophobic NCs, by directly interacting with the NC surface ligands through hydrophobic interactions, and when added in a concentration largely higher than its critical micelle concentration (CMC), it serves as a soft-template for the mesoporous structures. Upon removal of the volatile organic solvent, an optically transparent aqueous colloidal solution has been formed, confirming the success of the CTAB assisted NCs stabilization in water, with limited NC aggregation (Step II, Figure 2). The MSS growth, onto the CTAB-stabilized Cu_2−x_S NCs, has been carried out following a microemulsion approach [54] using ethyl acetate as an organic solvent, that is added in a volume ratio with the aqueous solution of nearly 1:15. TEOS, the silica precursor, is first dissolved in the ethyl acetate organic phase, then gradually hydrolyzed at the oil–water interface to allow surfactant–silicate assembly in the water phase and further condenses forming the silica network. The mesoporous structure is, thus, templated by CTAB together with ethyl acetate, surrounding the silica walls (Step III, Figure 2). 

**Figure 3:**
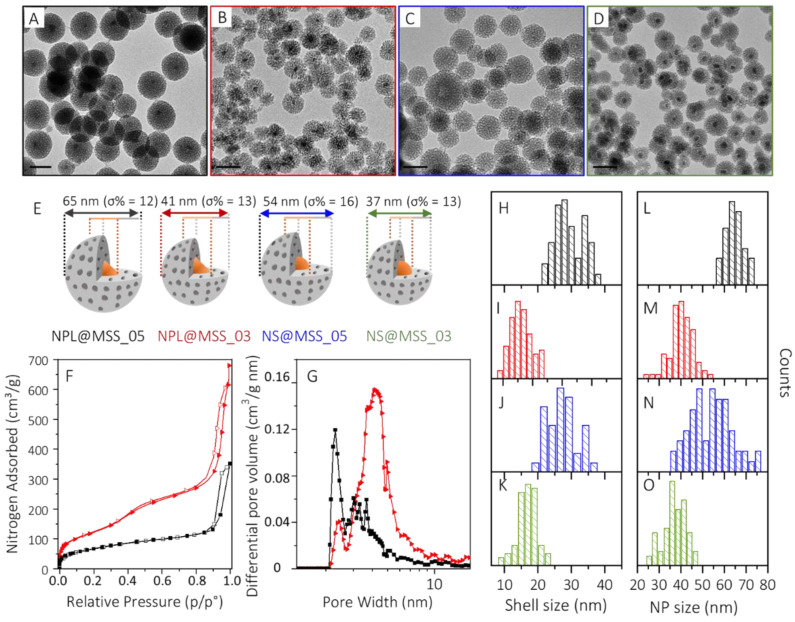
(**A**–**D**) TEM micrographs (scale bar 50 nm) of NPL@MSS_05 ((**A**), black frame), NPL@MSS_03 ((**B**), red frame), NS@MSS_05 ((**C**), blue frame) and NS@MSS_03 ((**D**), green frame) and (**E**) representative sketches of the core-shell NPs highlighting their size and size distribution, (**F**) nitrogen adsorption/desorption isotherm and (**G**) differential pore volume distribution of NPL@MSS_05 (black line) and NPL@MSS_03 (red line). Statistical analysis of the MSS (**H**–**K**) and core-shell NPs (**L**–**O**) size distribution as measured from TEM micrographs for NPL@MSS_05 (black bars), NPL@MSS_03 (red bars), NS@MSS_05 (blue bars) and NS@MSS_03 (green bars).

The application of such a synthetic scheme to coat the Cu_2−x_S NCs with an MSS requires conditions suited to prevent the degradation of the Cu_2−x_S NCs, as they are characterized by the high mobility of copper ions. Therefore, reaction conditions milder than those generally used for more robust NCs, usually involving pH > 11, a temperature around 60 °C and proceeding under air, have been applied [30,50,51,52,53]. Namely, the reactions have been carried out at pH 9 and at 40 °C, in a microemulsion saturated with an inert nitrogen atmosphere to limit the occurrence of Cu_2−x_S NCs dissolution and the concomitant loss of plasmonic properties. Similarly, bare MSN have been synthesized resulting in NPs with an average size of 45 nm (σ% = 16, Figure S3 in Supplementary Materials), prepared by using 0.5 mL of TEOS and 40 °C. In Table 1 the sample names, the CTAB and Cu_2−x_S NCs concentration and TEOS volume used for the preparation of the core-shell mesoporous structures are reported. The MSS thickness has been tuned by varying the volume of TEOS by 0.5 mL and 0.3 mL, at a fixed concentration of Cu_2−x_S (nearly 0.2 μM), resulting in the MSS_05 and MSS_03 MSS samples, respectively. The TEM micrographs of the core-shell nanostructures (Figure 3A–D) prepared by starting from triangular NPLs (Figure 3A,B) or spherical NSs (Figure 3C,D) cores, show, for both samples, the formation of a porous shell, homogenously coating the Cu_2−x_S NCs. 

MSS and core-shell nanostructures’ average size, measured by statistical analysis of the TEM micrographs are reported in Figure 3H–O, respectively, along with a schematic representation of each nanostructure, displayed in Figure 3E. The average diameter is 65 nm (σ% = 12) and 41 nm (σ% = 13) for NPL@MSS_05 and NPL@MSS_03, samples, respectively, and of 54 nm (σ% = 16) and 37 nm (σ% = 13) for NS@MSS_05 and NS@MSS_03. The corresponding shell thickness is 29 nm (σ% = 19) and 16 nm (σ% = 18) for NPL@MSS_05, NPL@MSS_03, and of 27 nm (σ% = 16) and 17 nm (σ% = 17) for NS@MSS_05 and NS@MSS_03, respectively. As expected, at increasing TEOS: Cu_2−x_S molar ratios, a thicker MSS is attained, with the shell thickness not critically affected by the Cu_2−x_S NC core geometry. Nitrogen absorption/desorption isotherm (Figure 3F) measured for the NPL@MSS_05 (Figure 3F, black line) and NPL@MSS_03 (Figure 3F, red line) along with the calculated differential pore volume distribution (Figure 3G), highlight the evident dependence of the mesoporous structure texture on the MSS thickness and, accordingly, on the amount of TEOS used to grow the MSS. Higher Brunauer–Emmett–Teller (BET) specific surface area (SSA) (450 ± 2 m^2^/g) and, concomitantly, larger pore volume (0.45 cm^3^/g) have been determined for NPL@MSS_03 compared to NPL@MSS_05 (BET SSA of 243 ± 2 m^2^/g and pore volume of 0.20 cm^3^/g). In particular, these nanostructures are characterized by dual mesoporosity, with one sharp peak centered around 2.3 nm and a broad distribution peak ranging from 2.8 nm to about 6 nm and 8 nm for NPL@MSS_05 and NPL@MSS_03, respectively. NPL@MSS_05 is characterized mainly by a narrower porosity (around 2.3 nm), while NPL@MSS_03 is prevalently constituted of larger pores, ranging from 2.8 nm to ca. 8 nm.

Similar values are also obtained for NS@MSS_05 and NS@MSS_03. The overall results suggest a correlation between the MSS porosity and the synthetic conditions. In particular, as reported by He et al. [55], higher CTAB/TEOS ratio leads to thinner shells characterized by larger porosity, higher SSA and total pore volume, while a lower CTAB/TEOS ratio leads to thicker shells, with narrower porosity, lower SSA and pore volume.

**Figure 4:**
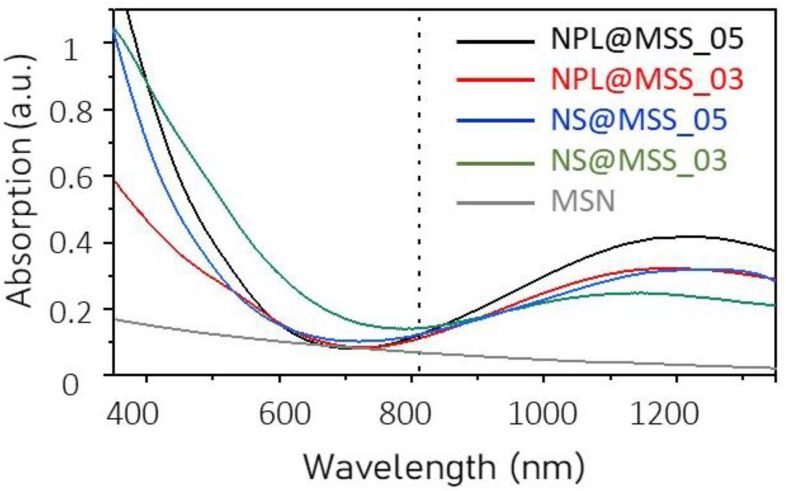
UV-Vis-NIR absorbance spectrum of each nanostructure along with the absorption spectrum of MSN (grey line). Dashed line at 808 nm, which is the wavelength of the CW laser light used for photothermal experiments.

The UV-Vis-NIR absorption spectra, reported in Figure 4, for samples NPL@MSS_05, NPL@MSS_03 and NS@MSS_05 and NS@MSS_03, still display the line profile characteristic of Cu_2−x_S NCs. A partial quenching of the LSPR band compared to the as-prepared Cu_2−x_S NCs is observed, that can be attributed to the susceptibility of the core to the external environment and to the modification of the surface chemistry, both reflecting on the plasmonic properties of the prepared systems. Dampening of the LSPR band is much more evident for the small NS (blue and green line Figure 4) rather than NPL (black and red line Figure 4) and for those core-shell NPs characterized by a thinner MSS (red and green line Figure 4). These results confirm the improved chemical stability of Cu_2−x_S NCs larger in size and the ability of a thicker and less porous MSS to better protect the core from the environment.

### 3.2. Photothermal Conversion Properties

To evaluate the photothermal characteristics of the prepared samples, each suspension has been purposely diluted to obtain the same absorption intensity (nearly 0.2) at 808 nm, which is the wavelength of the CW laser used to perform the light-to-thermal energy conversion experiments. Theoretical calculation (Figure S4), carried out to estimate the absorption and scattering cross-section of the plasmonic nanostructures, suggests that the scattering contribution to the absorption at this wavelength is not relevant either for the cores, NPLs and NSs [56], or for the entire core-shell nanostructures, in the size regime explored in this work. The limited impact of the MSS on the NIR-scattering is also confirmed by the UV-Vis-NIR absorption line profile recorded for the bare MSN suspension (grey line Figure 4F), that shows a negligible absorption intensity at 808 nm, for an MSN of nearly 45 nm. Although the 808 nm laser wavelength does not correspond to the maximum of the LSPR bands of the core-shell NPs, such a wavelength has been selected for the PT experiments, as it is commonly employed in clinics for various medical procedures [57,58]. Furthermore, the exploitation of the CW laser offers the possibility to irradiate the suspension for a prolonged time, and properly controlling the power density without damaging the nanostructures [59]. Conversely, high density femtosecond, picosecond or also nanosecond pulsed laser sources can induce detrimental phenomena, such as NCs melting or reshaping [60]. Uniform heating over large areas and overall temperature increase arise from the heat fluxes from individual NP that add up in a large and dense ensemble of NPs leading to high collective temperature [61].

Each suspension has been, thus, irradiated by CW pump beam at 808 nm lasting 60 s, using a laser beam density in the range of 6–38 W/cm^2^. The irradiation time and the power density have been varied for the fundamental investigation of the efficacy of the Cu_2−x_S NCs-based nanostructures in the light-to-thermal energy conversion. A high-resolution thermal camera has been exploited to identify both the spatial heating distribution and the temperature profile under lateral pumping laser irradiation. It is worth to note that no temperature increase has been observed for MSNs dispersed in an aqueous solution (data not reported). 

**Figure 5:**
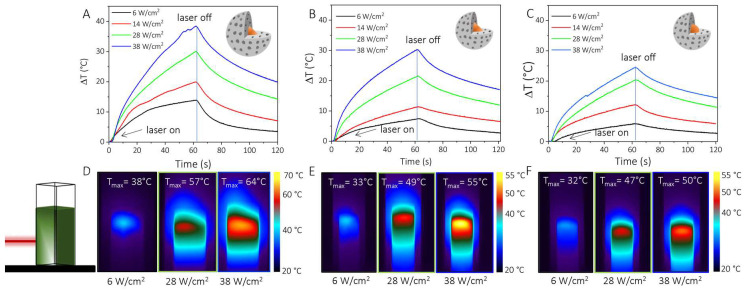
(**A**–**C**) Heating/cooling curves reporting the temperature increase versus time for NPL@MSS_05 (**A**), NPL@MSS_03 (**B**) NS@MSS_03 (**C**), irradiated by a CW laser light at 808 nm and 6 (black line) 14 (red line), 28 (green line) and 38 (blue line) W/cm^2^. The CW laser has been switched off after 60 s and the cooling rate was recorded, (**D–F**) pictures of the temperature distribution inside the cuvette, mapped by thermal camera at laser off.

**Figure 6:**
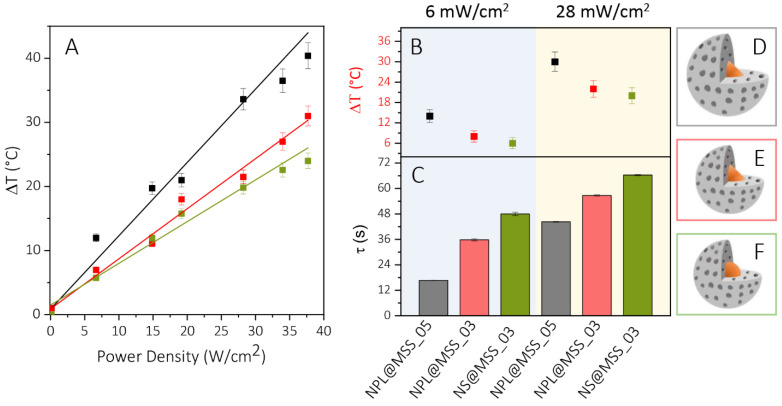
(**A**) Scatter plot and linear fitting of the temperature increase versus power density and (**B**) maximum temperature increase and (**C**) time constant values *τ* for NPL@MSS_05 (black), NPL@MSS_03 (red) and NS@MSS_03 (green). (**D**–**F**) Sketches of the core-shell nanostructures.

Figure 5 reports the heating/cooling curves at the different tested laser beam power densities for NPL@MSS_05 (Figure 5A), NPL@MSS_03 (Figure 5B) and NS@MSS_03 (Figure 5C) along with selected images acquired by the thermal camera after 60 s of irradiations, before shutting down the laser, for a laser beam density of 6 W/cm^2^, 28 W/cm^2^ and 38 W/cm^2^, respectively (Figure 5D–F). At an increasing laser beam power density, a pronounced increase in the temperature is registered for all the investigated nanostructures. The maximum temperature increase plotted versus the laser beam power density reported in Figure 6A for each sample has been linearly fitted, and the resulting R-squared values of nearly 0.98–0.99 suggest the goodness of the linear regression model to describe the dependence of temperature on the laser beam power density.

Based on the heating/cooling curves (Figure 5A–C), NPL@MSS_05 is a more effective PT system than NPL@MSS_03 and NS@MSS_03. For the former sample the measured temperature reaches 38 °C, 57 °C and 64 °C at 6 W/cm^2^, 28 W/cm^2^ and 38 W/cm^2^ power density, respectively, after 60 s of CW irradiation. The results illustrated in Figure 5 and Figure 6, discussed in terms of MSS thickness and textures, and Cu_2−x_S NC geometry, reveal a significantly larger temperature increase for the sample NPL@MSS_05, having an NPL core and a 29 nm thick shell, with a more compact structure. A slight increase is observed for NPL@MSS_03 when compared to NS@MSS_03 (Figure 6B), characterized by NPL and a spherical Cu_2−x_S core, respectively, and by a thinner MSS (ca. 17 nm). For the NS core, an increase in the MSS, as for the NS@MSS_05 sample (Figure S5 of the Supplementary Materials), featuring an MSS 27 nm-thick does not bring an additional temperature increase. TEM investigation of the samples after irradiation, reported in Figure S6 (Supplementary Materials), indeed, reveals the deterioration of the Cu_2−x_S cores in some core-shell NPs bearing NSs, suggesting a partial dissolution of NSs upon the irradiation condition exploited.

Conversely, the NPL@MSS_05 and NPL@MSS_03 samples remain almost unchanged (Figure S6, Supplementary Materials), with the dark spot ascribed to the Cu_2−x_S NPLs clearly still evident in the micrographs of both the samples, especially in the one characterized by a thinner MSS. The morphological characterization of the samples after laser light irradiation confirms the higher photostability of the NPLs over the NSs. These results suggest that smaller NSs are less suitable for PT applications having a limited photostability under the used laser beam power density. For NPL@MSS_05 and NPL@MSS_03 the photothermal stability has been also investigated after several cycles of irradiation, and almost negligible changes in the morphology have been detected, thus confirming the robustness of such nanostructures. Similarly, an almost unaltered PT response has been measured.

The time constant *τ* value, calculated from the cooling data after 60 s of laser light irradiation (Figure S7 in Supplementary Materials), is reported in Figure 6C for 6 W/cm^2^ and 28 W/cm^2^ power density. Their values are nearly 17 s for NPL@MSS_05 and increases to 37 s for NPL@MSS_03 at 6 W/cm^2^ reaching, in both cases, a higher value at 28 W/cm^2^. According to the Roper model [24,48], the smaller the time constant τ the larger is the heat transfer efficiency. As a consequence, the PT conversion efficiency, calculated for 6 W/cm^2^ laser light power density, results higher than 20% for NPL@MSS_05, while it drops to nearly 5% for NPL@MSS_03. Moreover, efficiency decreases as the power density increases. 

This study highlights the correlation between PT conversion efficiency and MSS properties under CW laser light irradiation, in agreement with a previous report [24]. Considering that a low volume of TEOS (at fixed Cu_2−x_S concentration) provides a thinner shell and, at the same time, a higher porosity in the mesoporous structures and since the thermal conductivity of silica decreases with increasing porosity, the lower PT conversion efficiency calculated for NPL@MSS_03 can be reasonably ascribed to a heat transfer rate taking place in this sample, which features a higher porosity, lower than that occurring in NPL@MSS_05.

Based on the results of the light-to-thermal energy conversion experiments, the NPL@MSS_05 sample has been identified as for the most promising candidate for future in vitro and in vivo applications. DLS and ζ- potential measurements have been carried out to evaluate the effective colloidal stability in aqueous media and the surface charge density for the NPL@MSS_05 sample after treatment with HCl solution to remove the CTAB surfactants from the mesoporous structures and compare them with those measured for the bare MSN sample. 

As shown in Table 2, the hydrodynamic diameters are in good agreement with the TEM characterization of the corresponding samples (Figure 3B and Figure S3 in Supplementary Materials). As expected, the MSS exposing the silanol groups and similarly for the MSN, a negative ζ- potential value has been measured whose absolute value highlights good colloidal stability. 

**Figure 7:**
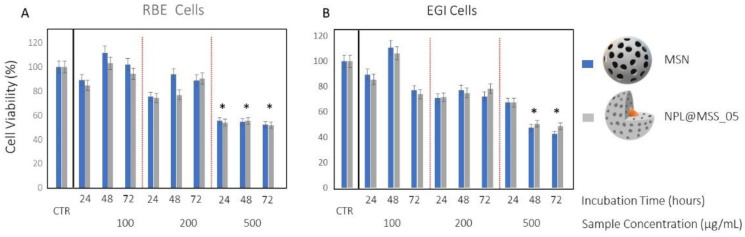
Cell viability tests on RBE (**A**) and EGI (**B**) cell lines treated with MSS and NPL@MSS_05 for 24. 48 and 72 h at the concentration of 100, 200 and 500 µg mL^−1^. Control: untreated cells. * *p* < 0.05.

It is worth noting that the silica surface is, in general, characterized by a promising chemical versatility, and different functionalities; amine groups could be easily grafted via reactions involving bifunctional siloxane molecules, thus offering the possibility to manipulate the charge density from a negative to a much more positive value. Amine groups can also act as anchoring points for the covalent binding of specific molecules or polymers. A preliminary study (Figure 7) carried out to investigate the in vitro cytotoxicity of the mesoporous silica-based nanostructures has enabled the evaluation of the degree of sample biocompatibility for their future use as nanocarriers for PT therapy and drug delivery. Two different cell lines, namely the human intrahepatic cholangiocarcinoma RBE cells and the human extrahepatic cholangiocarcinoma EGI-1 cells, have been used for the in vitro toxicity assay. These cell lines have been selected since cholangiocarcinoma (CCA) is a highly aggressive and chemoresistant desmoplastic cancer; therefore, the exploitation of the PT therapy is expected to represent an efficacious adjuvant therapeutic approach for its treatment [62]. 

Cells have been incubated with MSS and NPL@MSS_05 at concentrations ranging from 100 μg mL^−1^ to 500 μg mL^−1^ for 24, 48 and 72 h. For both samples and all investigated incubation times, no statistically significant effects on cell viability have been observed when the lowest tested concentrations (100 and 200 µg/mL) have been administrated to the two cell lines. However, even at the highest tested concentration, 500 µg/mL, a percentage of survival cells above 50% has been always observed upon treatment of both the RGB cells and EGI cells with the two mesoporous silica-based samples at 24, 48 and 72 h, indicating a very limited reduction in cell viability. The obtained results have also proved that the presence of the Cu_2−x_S NCs in the core of the mesoporous silica based-nanostructures does not induce any additional cytotoxic effect if compared with bare MSNs.

## 4. Conclusions

Here, mesoporous silica-coated Cu_2−x_S NC samples, featuring different core geometry, average size, MSS thickness and textures have been synthesized by solution approaches, and have been thoroughly characterized by morphological, spectroscopic investigation to be further used as nanoheaters for PT therapy.

The developed microemulsion approach, performed under an inert atmosphere, low temperature (40 °C) and slightly basic condition (pH = 9), was used for the growth of the MSS; it resulted in nanostructures characterized by a Cu_2−x_S NCs core homogenously coated by the MSS. Tuning the thickness of the MSS, by using a variable amount of TEOS at fixed Cu_2−x_S NCs and CTAB concentrations, concomitantly promoted change of the mesoporous structures texture. In particular, a large volume of TEOS provided a thicker shell with a more compact structure, while lower a TEOS volume resulted in a thinner and more porous structures. For fundamental analysis of the light-to-heat conversion each synthesized sample has been irradiated by CW laser light at 808 nm, using a power density in the range of 6 to 38 W/cm^2^. The thermoplasmonic experiments have pointed out the limited photostability of small Cu_2−x_S NSs under the tested irradiation condition, while the NPLs have resulted in more robust plasmonic cores. Moreover, the larger temperature increase measured for NPL@MSS_05 than NPL@MSS_03, the shorter time constant (*τ*) and the higher PT efficiency have suggested an enhanced and faster heat flow from the plasmonic core coated with MSS tens of nanometers thick, and characterized by a more compact texture, rather than by the thinner and highly porous MSS.

## Figures and Tables

**Table 1:** Summary of type of samples, concentration of cetyltrimethylammonium bromide (CTAB) and Cu_2−x_S NCs and tetraethylortosilicate (TEOS) volume used for the synthesis of the core-shell NPs using Cu_2−x_S nanospheres (NS) and nanoplates (NPL) as cores.

Sample Name	CTAB (mM)	Cu_2−x_S (μM)	TEOS (mL)
NPL@MSS_05	5	0.2	0.5
NPL@MSS_03	5	0.2	0.3
NS@MSS_05	5	0.2	0.5
NS@MSS_03	5	0.2	0.3

**Table 2:** Size analysis by dynamic light scattering and ζ- potential measurement for MSN and NPL@MSS_05 core-shell nanostructures.

Sample Name	Size by DLS (nm)	PDI	ζ Potential Value (mV)
MSN	51	0.38 ± 0.05	−31.2 ± 6.8
NPL@MSS_05	93	0.30 ± 0.02	−27.3 ± 6.0

## Data Availability

The data presented in this study are available on request from the corresponding author.

## Supplementary Materials

The following are available online at https://www.mdpi.com/2079-4991/12/15/2545/s1, Figure S1: Set-up used for the photothermal experiments; Figure S2: Plasmonic features as measured from UV-Vis-NIR absorption spectra and Energy Dispersive X-ray spectrum with table reporting the atomic percentage and stoichiometry of Cu_2−x_S NPL and NS samples, UV-Vis NIR absorption spectrum of Cu_2−x_S before and after ligands treatment; Figure S3: TEM characterization of bare MSN; Figure S4: Computational estimation of the scattering and absorption contribution to the extinction cross section; Figure S5: Heating/cooling curves for NS@MSS_05 sample; Figure S6: TEM characterization of the synthesized core-shell NPs after irradiation for 60 s with a CW laser light at 808 nm and 6 W/cm^2^ power density; Figure S7: Plot of the negative reciprocal slope of *ln*(*θ*) versus *t,* used for measuring time constant *τ*.
