# Supplementary material for: NIR-Absorbing Mesoporous Silica-Coated Copper Sulphide Nanostructures for Light-to-Thermal Energy Conversion
Source: Nanomaterials (Basel). 2022 Jul 24;12(15):2545. doi: 10.3390/nano12152545 (PMC9330451; doi:10.3390/nano12152545)
Supplement: Supplementary file 1 [file nanomaterials-12-02545-s001.zip › nanomaterials-1815352-supplementary.pdf]

nanomaterials

MDPI

Supplementary Materials

NIR-Absorbing Mesoporous Silica-Coated Copper Sulphide Nanostructures for Light-to-Thermal Energy Conversion

Elisabetta Fanizza [1,2,*], Rita Mastrogiacomo [1,2], Orietta Pugliese [1], Alexa Guglielmelli [3,4], Luciano De Sio [4,5], Rachele Castaldo [6], Maria Principia Scavo [7], Mariangela Giancaspro [1,2], Federica Rizzi [1,2], Gennaro Gentile [6], Fabio Vischio [2], Livianna Carrieri [7], Ilaria De Pasquale [2], Giacomo Mandriota [2], Francesca Petronella [8], Chiara Ingrosso [2], Marino Lavorgna [9], Roberto Comparelli [2], Marinella Striccoli [2], Maria Lucia Curri [1,2] and Nicoletta Depalo [2,*]

[1] Department of Chemistry, University of Bari, A. Moro, Via Orabona 4, 70126 Bari, Italy; r.mastrogiacomo@ba.ipcf.cnr.it (R.M.); oriettapugliese44@gmail.com (O.P.); mariangela.giancaspro@uniba.it (M.G.); federica.rizzi@uniba.it (F.R.); marialucia.curri@uniba.it (M.L.C.)
[2] CNR-IPCF Bari Division, Via Orabona 4, 70125 Bari, Italy; f.vischio@ba.ipcf.cnr.it (F.V.); i.depasquale@ba.ipcf.cnr.it (I.D.P.); g.mandriota@ba.ipcf.cnr.it (G.M.); c.ingrosso@ba.ipcf.cnr.it (C.I.); r.comparelli@ba.ipcf.cnr.it (R.C.); m.striccoli@ba.ipcf.cnr.it (M.S.)
[3] Department of Physics, NLHT-Lab, University of Calabria, Via Ponte P. Bucci, Cubo 33C, 87036 Rende , Italy; alexa.guglielmelli@unical.it
[4] CNR-NANOTEC-Institute of Nanotechnology, Via Ponte P. Bucci, Cubo 33C, 87036 Rende , Italy; luciano.desio@uniroma1.it
[5] Department of Medico-Surgical Sciences and Biotechnologies, Research Center for Biophotonics, Sapienza University of Rome, Corso della Repubblica 79, 04100 Latina, Italy
[6] CNR-IPCB, Via Campi Flegrei 34, 80078 Pozzuoli , Italy; rachele.castaldo@ipcb.cnr.it (R.C.); gennaro.gentile@ipcb.cnr.it (G.G.)
[7] Personalized Medicine Laboratory, National Institute of Gastroenterology "S. De Bellis", Via Turi 21, 70013 Castellana Grotte, Italy; maria.scavo@irccsdebellis.it (M.P.S.); livianna.carrieri@irccsdebellis.it (L.C.);
[8] CNR-IC, Via Salaria Km 29.300, 00015 Monterotondo, Italy; francesca.petronella@ic.cnr.it
[9] CNR-IPCB, Piazzale E. Fermi 1, 80055 Portici, Italy; marino.lavorgna@cnr.it
* Correspondence: elisabetta.fanizza@uniba.it (E.F.); n.depalo@ba.ipcf.cnr.it (N.D.)

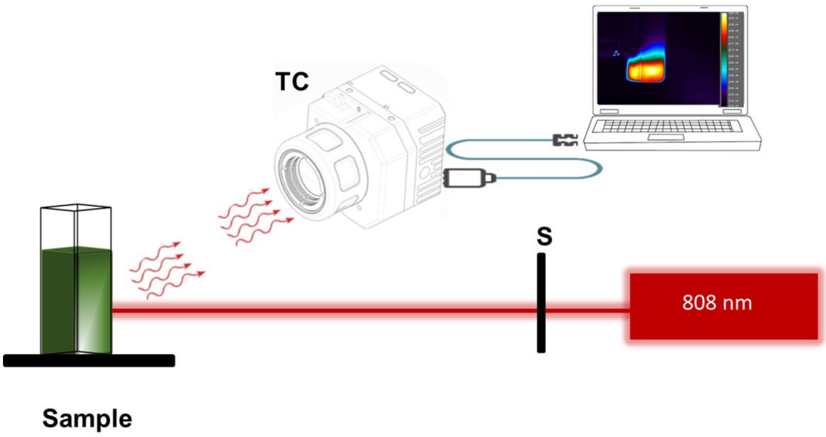

Figure S1. Sketch of the optical set-up used for the photothermal investigation. A quartz cuvette (Sample) filled with the samples (1 mL) was irradiated with CW diode laser light emitting at 808 nm. The laser beam is activated by an optical shutters (S). The spatial-temporal temperature profiles are detected with a high-resolution thermal camera (TC).

A

Sample	NPL	NS
λ_{LSPR} (nm)	1368	1263
ω_p (eV)	2.65	2.73
γ (eV)	0.66	0.60
N_h (cm^{-3})	4.00E+21	4.23E+21
Size (nm)	14 σ%=12	6 σ%=11
$n_{h,NC}$	8575	478
$Cu_{\%}$ (%)	9.4	10.0
$Cu_{2-x}S$	$Cu_{1,81}S$	$Cu_{1,80}S$
ε_{ext} (ω_{LSPR}) ($M^{-1}cm^{-1}$)	1.3E+07	1,8E+07

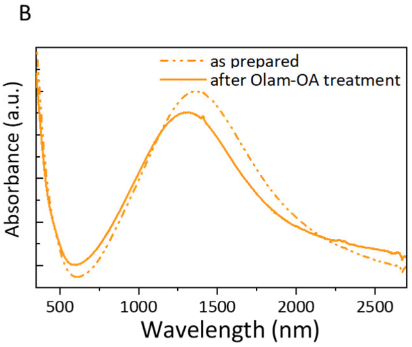

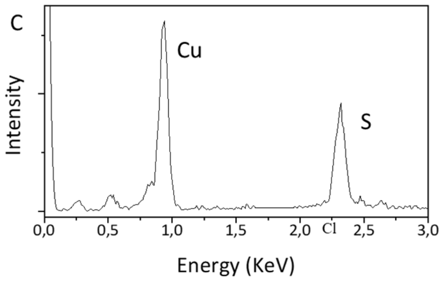

D

Sample	Cu atomic%	S atomic %	stoichiometry
$Cu_{2-x}S$ NPL	22.51	12.22	1.84
$Cu_{2-x}S$ NS	12.60	7.06	1.79

Figure S2. (A)Table of the plasmonic features as λ_{LSPR} (maximum wavelength of the LSPR band), γ (full width at half maximum), determined from the UV-Vis NIR absorbance spectra, ω_p (bulk plasmon band), N_h (hole carrier density) and ε_{ext}(extinction coefficient) as well as copper deficiency, theoretically determined adopting the Mie Theory; (B) UV-Vis-NIR absorbance spectra of the Cu2-xS nanoplate (NPL) colloidal solution before and after the treatment with fresh ligands Oleyl amine (Olam) and oleic acid (OA); (C, D) Energy Dispersive X-ray (EDX) spectrum of $Cu_{2-x}S$ nanocrystals and table reporting the atomic percentage of copper and sulphur and the corresponding stoichiometry for $Cu_{2-x}S$ NPL and spherical nanoparticles (NS) colloidal solution drop casted on silicon substrate, as measured by EDX analysis.

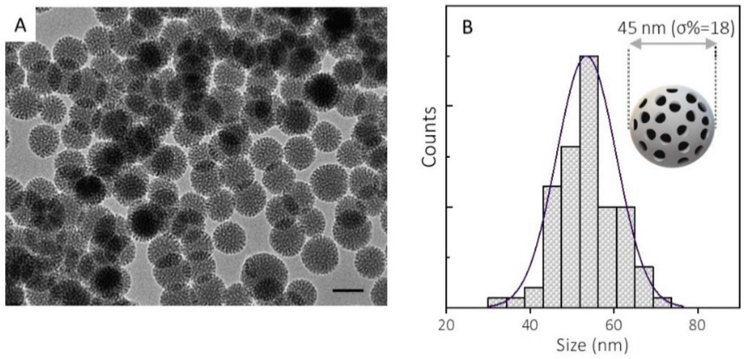

Figure S3. TEM micrograph (A, scale bar 50 nm) and statistical analysis (B) of the diameters of mesoporous nanoparticles (MSN).

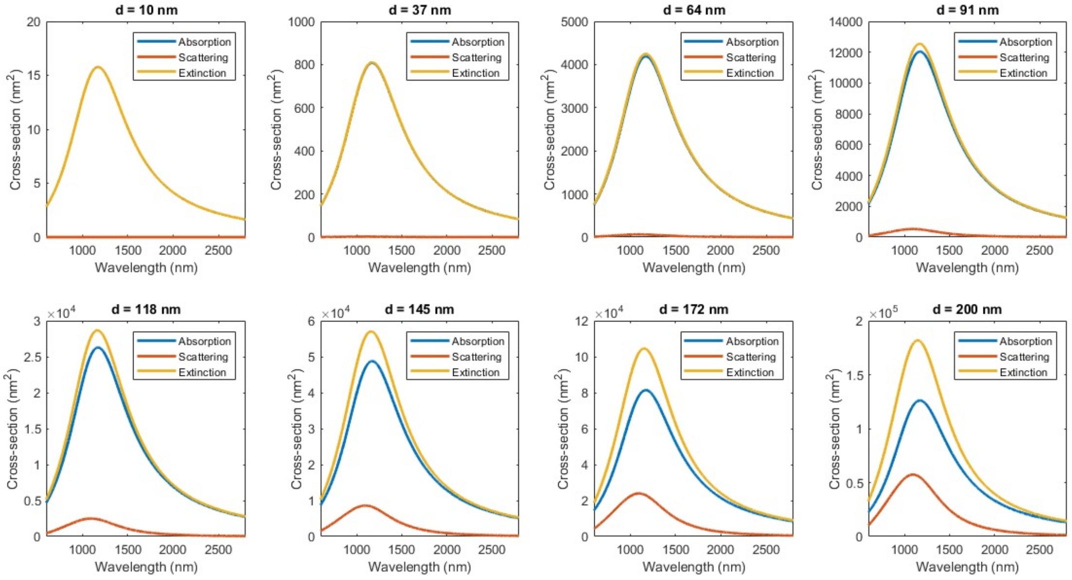

Figure S4. Computational estimation of the scattering (red line), absorption (blue line) and extinction (yellow line) cross-section contribution depending on the plasmonic NC diameter, d. The scattering cross section starts to become relevant for NC diameter up to ninety nm, while below this threshold size the main contribution to the extinction cross section comes from the absorption.

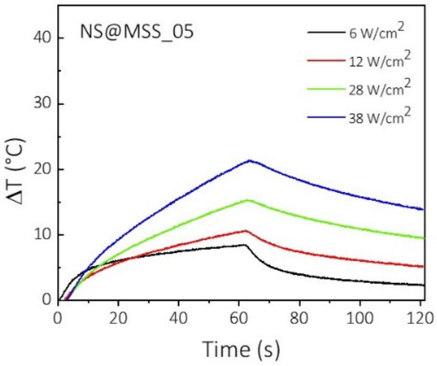

Figure S5. Heating/cooling curves reporting the temperature increase versus time for NS@MSS_05

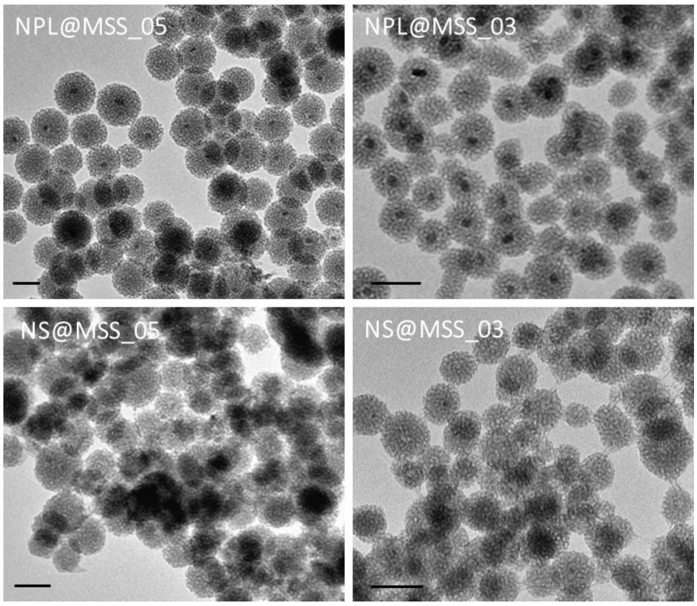

Figure S6. TEM micrographs (scale bar 50 nm) of core-shell structures NPL@MSS_05, NPL@MSS_03, NS@MSS_05, NS@MSS_03 after irradiation with a CW laser beam at 808 nm at increasing power density.

$$\theta = \frac{T(t) - T_{sur}}{T_{max} - T_{sur}} \qquad -ln\theta = \frac{t}{\tau}$$

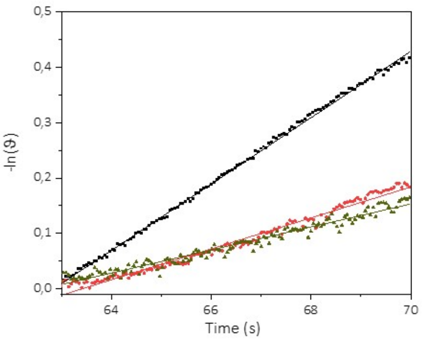

Figure S7. Plot of the negative reciprocal slope of ln(θ) versus *t* using temperature versus time data recorded during cooling after the CW laser beam 808 nm switched off (power density 6 W/cm^2) for NPL@MSS_05 (black line), NPL@MSS_03 (red line) and NS@MSS_03 (green line).
